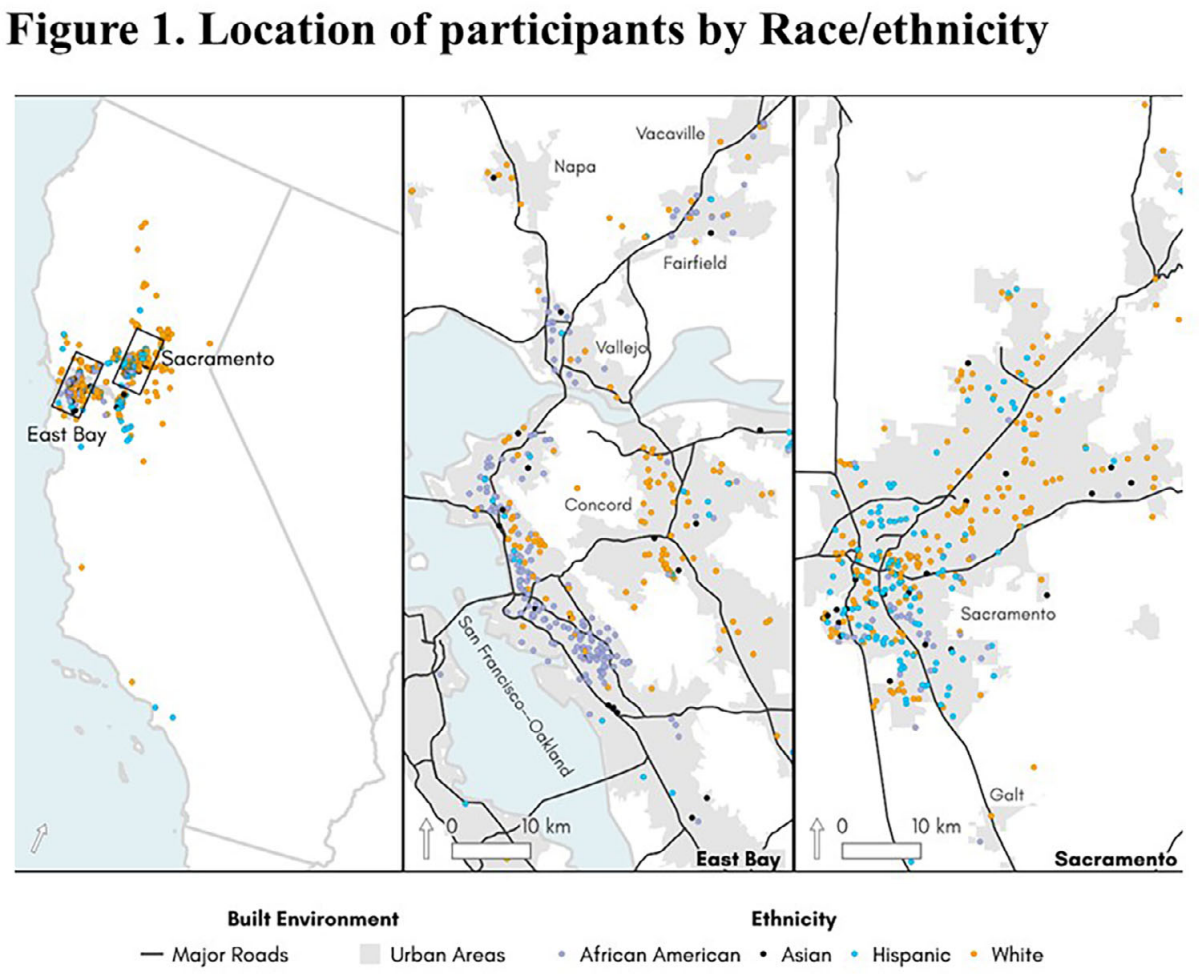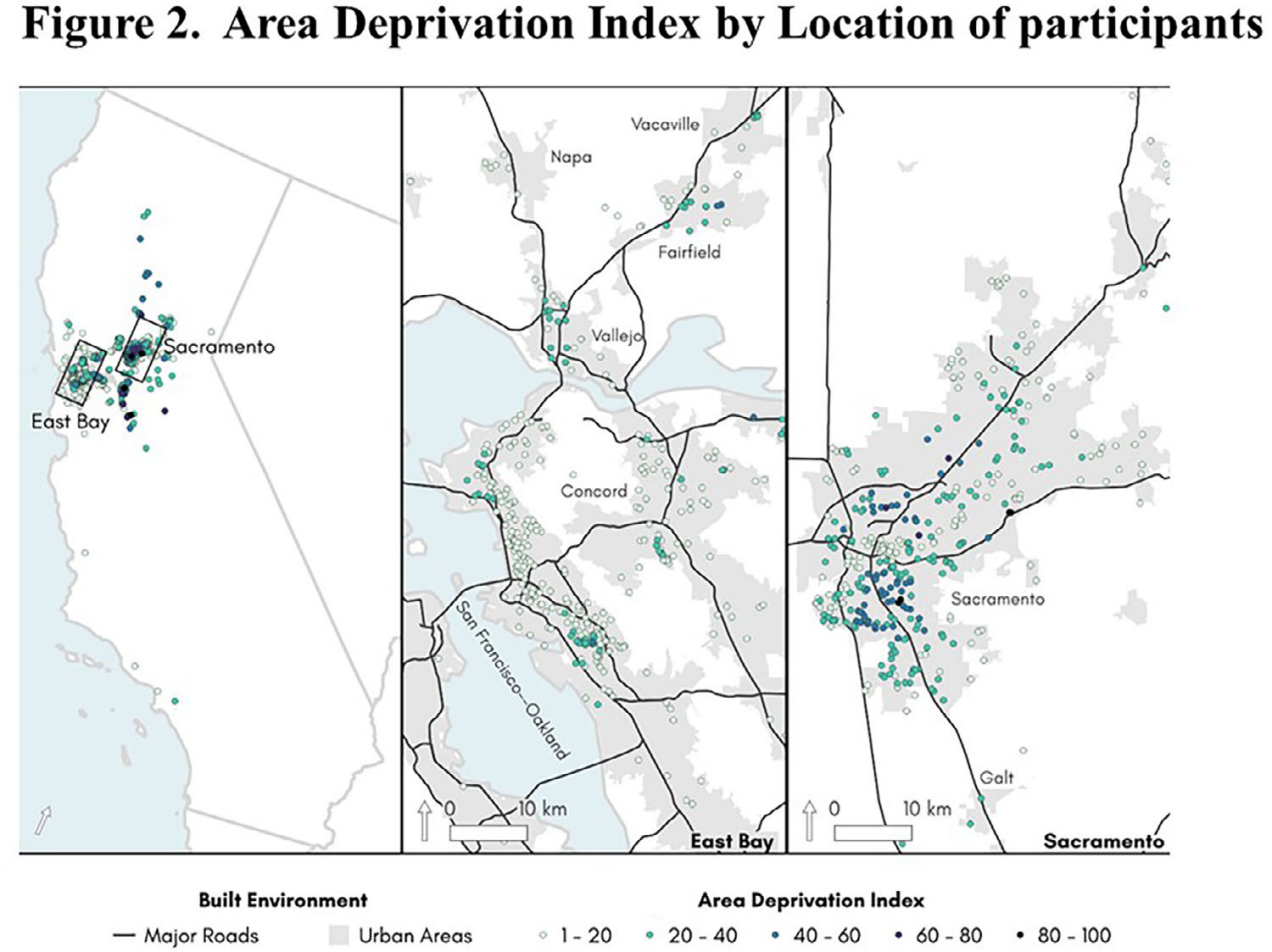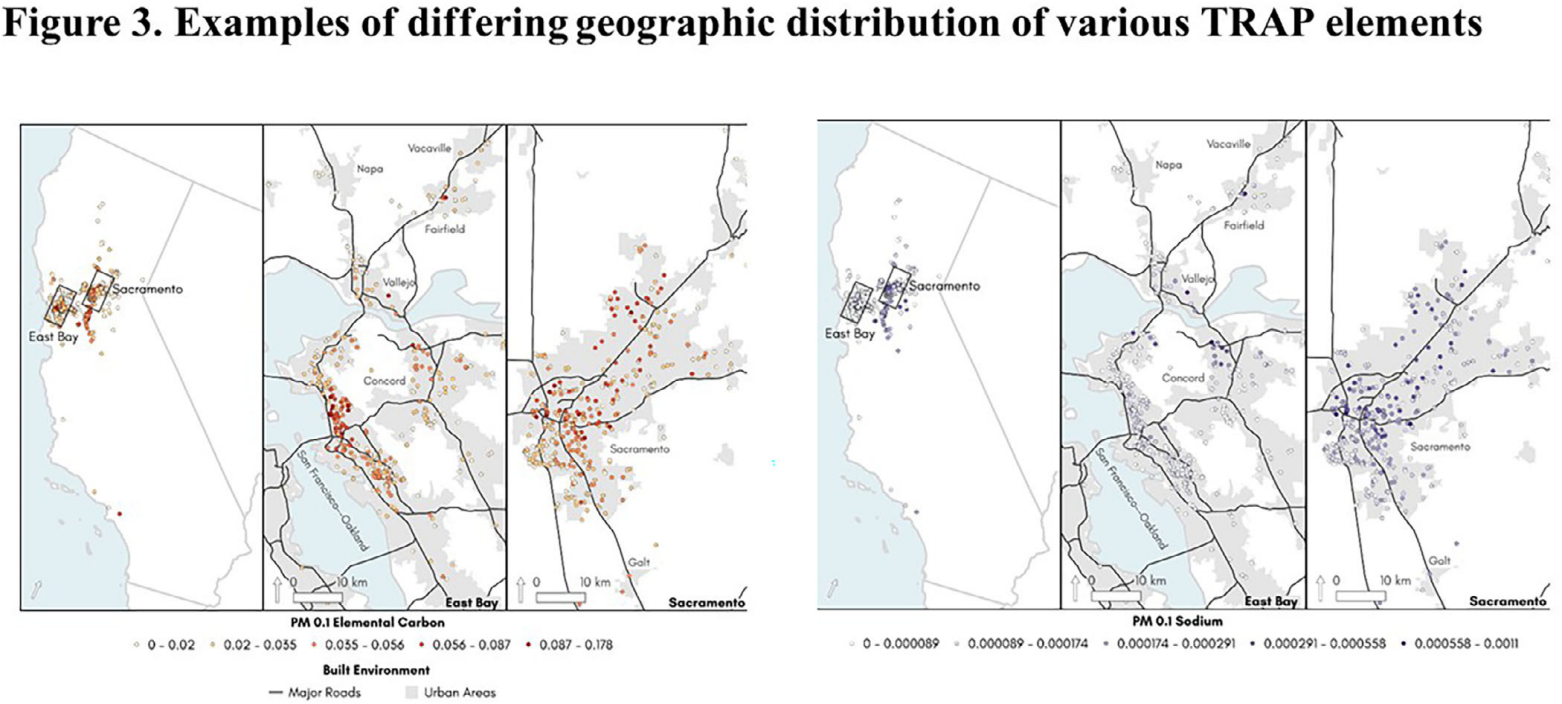# Characterizing Exposure to Traffic‐Related Air Pollution in a Diverse Alzheimer’s Disease Research Center Cohort

**DOI:** 10.1002/alz.092071

**Published:** 2025-01-09

**Authors:** Charles Decarli, Sarah Tomaszewski Farias, Michele Tobias, Danielle J. Harvey, Randy Carney, Anthony Wexler, Keith Bein, Mike Kleeman, Pamela Lein, Oanh L. Meyer

**Affiliations:** ^1^ University of California, Davis, Sacramento, CA USA; ^2^ University of California, Davis, Davis, CA USA; ^3^ University of California, Davis School of Medicine, Sacramento, CA USA; ^4^ UC Davis, Davis, CA USA

## Abstract

**Background:**

Most studies on the associations between traffic related air pollution (TRAP), cognition, and dementia focus on particulate matter (PM) 2.5, are cross‐sectional and based on non‐Hispanic White (NHW) individuals. Less is known about how exposure to ultrafine PM (UFPM) affects cognition in racially/ethnically diverse cohorts. The UC Davis Alzheimer’s Disease Research Center (UCD ADRC) recently launched a study on TRAP that will assess the impact of UFPM on cognition, brain imaging and neuropathology. This abstract describes the broad geographic location, varying TRAP exposure, and demographic diversity of the cohort.

**Method:**

A total of 985 adults, average age of 81.3 ± 8.2 years, 62.6% female and consisting of 44% NHW, 24% Hispanic/Latino, 26.4% Black/African American, and approximately 4% other races/ethnicities had physical addresses geocoded to identify geographic coordinates; 29% of participants were cognitively normal, 18% had questionable cognitive impairment, 23% had mild cognitive impairment, and 30% had dementia. Detailed neuropsychological assessments and MRI were conducted annually along with collection of blood samples and data regarding social cultural variables. Median exposure of nearly 30 different TRAP measures including PM ranging in size from 0.1 to 10 µm measured over the years 2000‐2019 were calculated. Individual exposures were determined over an average of 4.4 ± 4.7 years of participant follow‐up.

**Result:**

Participants lived in 28 counties throughout northern California with major clusters in the East Bay, Vallejo, Fairfield, Vacaville, Sacramento, and Central Valley south of Sacramento. We found highly significant differences in ethnoracial distribution according to county designation (Figure 1; p < 0.001; Fisher’s exact) and in both state and national Area Deprivation Index scores (ADI; Figure 2; p <0.0001, one‐way ANOVA). ADI varied significantly with ethnoracial background with Hispanic/Latino participants having the highest ADI at 29 ± 1. MANOVA analysis found significant (p <0.0001) geographic differences in 0.1 µm sized particles (e.g. Figure 3 for sodium and elemental carbon).

**Conclusion:**

Preliminary analysis indicates significant differences in location, ethnoracial background, ADI, and TRAP exposure amongst UCD ADRC participants. This heterogeneity will allow comprehensive evaluation of the clinical impact of TRAP exposure on cognition among diverse individuals.